# Supplementary material for: DMRforPairs: identifying Differentially Methylated Regions between unique samples using array based methylation profiles
Source: BMC Bioinformatics. 2014 May 15;15:141. doi: 10.1186/1471-2105-15-141 (PMC4046028; doi:10.1186/1471-2105-15-141)
Supplement: Additional file 2 — DMRforPairs output for the comparison of A431-MCF7 and NA17018-NA17105. Please start from the HTML files in each folder. Available via the BMC Bioinformatics website. [file 1471-2105-15-141-S2.zip › 1394847754114233_MOESM2_ESM/NA17105M_NA17018F/figures/21849.pdf]

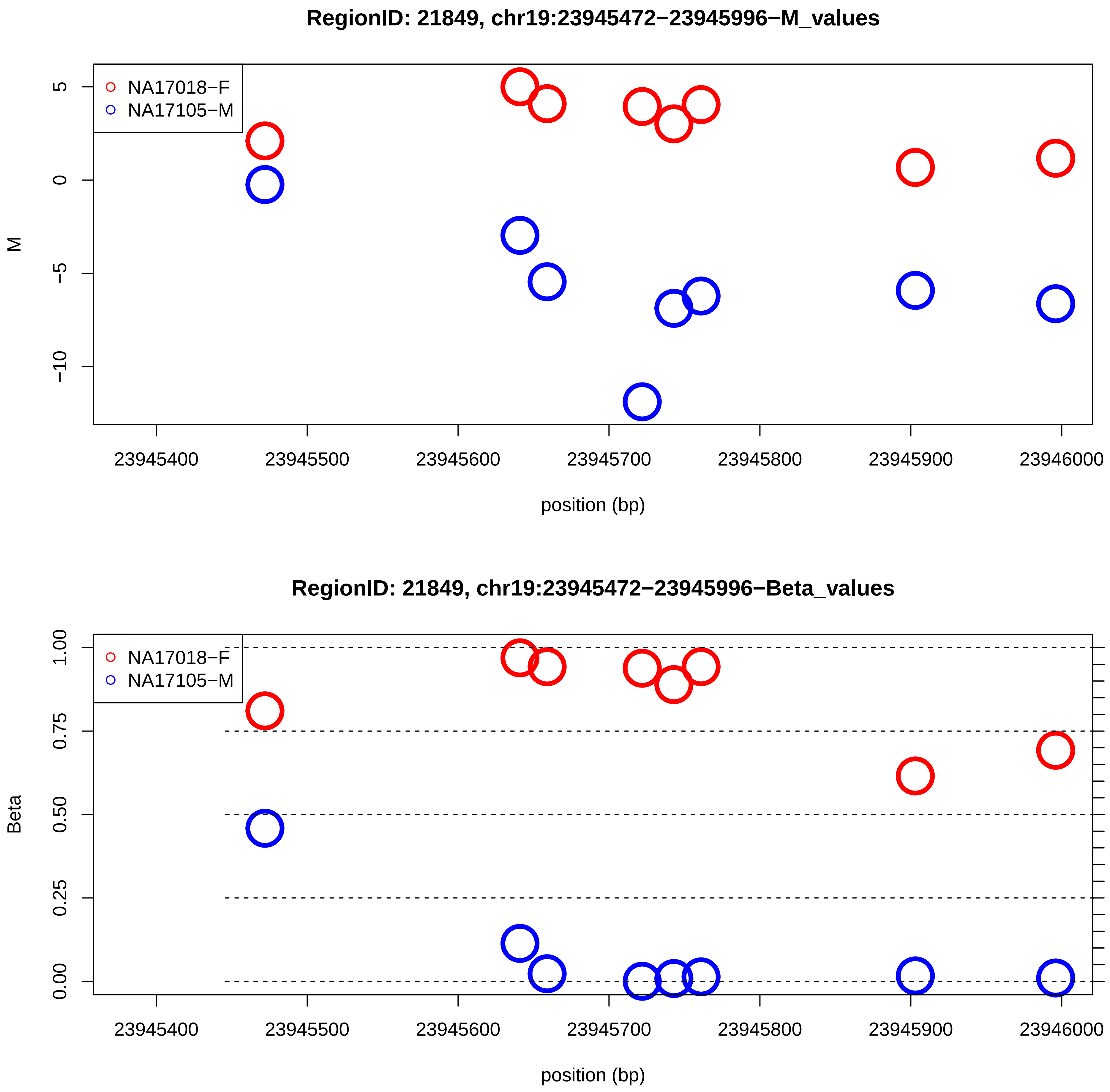
RegionID: 21849, chr19:23945472−23945996−M_values
NA17018−F
NA17105−M
M
5
0
−5
−10
23945400
23945500
23945600
23945700
23945800
23945900
23946000
position (bp)
RegionID: 21849, chr19:23945472−23945996−Beta_values
NA17018−F
NA17105−M
Beta
1.00
0.75
0.50
0.25
0.00
23945400
23945500
23945600
23945700
23945800
23945900
23946000
position (bp)

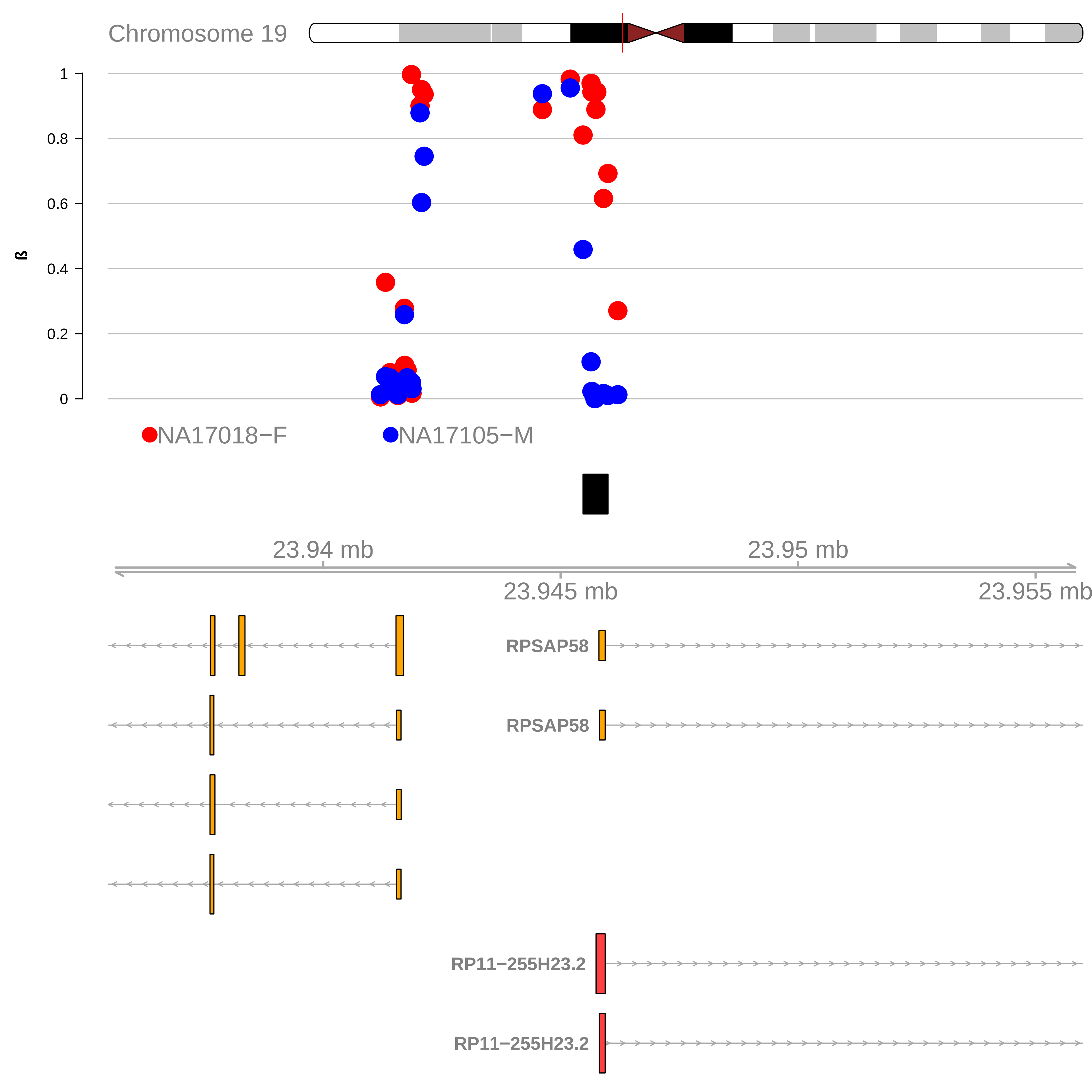

Chromosome 19
1
0.8
0.6
0.4
0.2
0
β
NA17018−F
NA17105−M
23.94 mb
23.95 mb
23.945 mb
23.955 mb
RPSAP58
RPSAP58
RP11−255H23.2
RP11−255H23.2
